# 

**Published:** 1899-05

**Authors:** 


### BOOKS RECEIVED.

Urinary Analysis and Diagnosis by Microscopical and Chemical Examinations. By Louis Heitzmann, M. D., New York. One volume, 270 pages, 8vo, 108 original illustrations. Price, muslin, $2.00 net. New York: William Wood & Company. 1899.

Chemistry: General, Medical and Pharmaceutical, including the Chemistry of the U. S. Pharmacopœia. By John Attfield, F. R. S. New (sixteenth) edition. In one royal 12mo volume of 784 pages, with eighty-eight illustrations. Cloth, $2.50 net. Philadelphia and New York: Lea Brothers & Company.

The International Medical Annual and Practitioner's Index. A work of reference for modern practitioners. Seventeenth year. New York: E. B. Treat, 241–243 W. 23d street. 1899.

Annual and Analytical Cyclopedia of Practical Medicine. By Charles E. de M. Sajous, M. D., and 100 associate editors. Assisted by corresponding editors, collaborators and correspondents. Illustrated with chromo-lithographs, engravings and maps. Volume III. Dislocations to Infantile Myxedema. Philadelphia, New York and Chicago: F. A. Davis Company. 1899.

The Serum Diagnosis of Disease. By Richard Cabot, M. D., Physician to Out-patients, Massachusetts General Hospital. Small 8vo, pp. vii—154. New York: William Wood & Company. 1899.

The Pathology and Treatment of Sexual Impotence. By Victor G. Vecki, M. D. From the author's second German edition, revised and rewritten. Duodecimo, pp. 291. Price, $2.00 net. Philadelphia: W. B. Saunders. 1899.

An Essay on the Nature and the Consequences of Anomalies of Refraction. By F. C. Donders, M. D., Late Professor of Physiology and Ophthalmology in the University of Utrecht. Revised and edited by Charles A. Oliver, M. D., one of the attending physicians to the Wills' Eye Hospital, etc. Illustrated. Price, $1.25. Philadelphia: P. Blakiston's Son & Company. 1899.

Retinoscopy (or shadow test) in the Determination of Refraction at One Meter Distance, with the Plane Mirror. By James Thorington, M. D., Adjunct Professor of Diseases of the Eye in the Philadelphia Polyclinic and College for Graduates in Medicine, etc. Third edition, revised and enlarged. Forty-three illustrations. Price, $1.00. Philadelphia: P. Blakiston's Son & Company. 1899.

Surgical Nursing. By Bertha M. Voswinkel, Graduate of Episcopal Hospital, Philadelphia; late nurse-in-charge of Children's Hospital, Columbus, O. Second edition, revised and enlarged, with 112 illustrations. Price, $1.00. Philadelphia: P. Blakiston's Son & Company. 1899.

The Anatomy of the Central Nervous System of Man and of Vertebrates in General. By Prof. Ludwig Edinger, M. D., Frankfort-on-the-Main. Translated from the Fifth German Edition by Winfield S. Hall, Ph. D., M. D., Professor of Physiology in the Northwestern Medical School, Chicago. Assisted by Philo Leon Holland, M. D., Instructor in Clinical Neurology in the Northwestern University Medical School, and Edward P. Carleton, B. S., Demonstrator of Histologic Neurology in the Northwestern University Medical School, Chicago. Illustrated with 258 engravings; 6½ x 9½ inches; pp. xi. - 446. Extra cloth, $3.00. The F. A. Davis Co., Publishers, 1914–16 Cherry street, Philadelphia.

Atlas of External Diseases of the Eye. By Dr. O. Haab, of Zurich. Edited by G. E. de Schweinitz, M. D., Professor of Ophthalmology, Jefferson Medical College, Philadelphia. With 100 colored illustrations. Price, $3.00 net. Philadelphia: W. B. Saunders. 1899.

Practical Materia Medica for Nurses, with an Appendix. Containing Poisons and their Antidotes, with Poison Emergencies, Mineral Waters, Weights and Measures, etc., etc. By Emily A. M. Stoney, Graduate of the Training School for Nurses, Lawrence, Mass., etc., etc. Duodecimo, pp. 306. Price, $1.50 net. Philadelphia: W. B. Saunders. 1899.

The Principles of Bacteriology. A practical manual for students and physicians. By A. C. Abbott, M. D., Professor of Hygiene and Director of the Laboratory of Hygiene, University of Pennsylvania, Philadelphia. New (fifth) edition, enlarged and thoroughly revised. Duodecimo, 585 pages, 109 illustrations, of which twenty-six are colored. Cloth, $2.75 net. Philadelphia and New York: Lea Brothers & Company.

